# PROMIS-29 and EORTC QLQ-C30: an empirical investigation towards a common conception of health

**DOI:** 10.1007/s11136-022-03324-7

**Published:** 2023-01-09

**Authors:** Claudia Hartmann, Felix Fischer, Christoph P. Klapproth, Robert Röhle, Matthias Rose, Maria M. Karsten

**Affiliations:** 1 Department of Psychosomatic Medicine, Center of Internal Medicine and Dermatology, Charité - Universitätsmedizin Berlin, Charitéplatz 1, 10117 Berlin, Germany; 2 Department of Gynecology With Breast Center, Charité University Hospital Berlin, Berlin, Germany; 3 Department of Quantitative Health Sciences, University of Massachusetts Medical School, Worcester, USA

**Keywords:** PROMIS-29, EORTC QLQ-C30, Health-related quality of life, Conceptualization of health, Comparable outcome

## Abstract

**Purpose:**

The assessment of health-related quality of life (HRQOL) measured via patient-reported outcomes (PROs) is a key component in clinical trials and increasingly used in clinical routine worldwide. Two PRO measures (PROMs) that share the same definition of health and report outcomes on a comparable T-metric anchored to general population samples are the PROMIS-29 and the EORTC QLQ-C30. In this study, we investigate the empirical agreement of these underlying concepts.

**Methods:**

We collected PROMIS-29 and EORTC QLQ-C30 data from 1,478 female patients at a breast cancer outpatient centre. We calculated descriptive statistics and correlations between the subscales of both instruments. We performed exploratory (EFA) and confirmatory factor analysis (CFA) in randomly split subsamples in order to assess the underlying psychometric structure of both instruments.

**Results:**

The cohort (mean age = 47.4, ± 14.49) reported comparable mean HRQOL scores between the corresponding subscales of both instruments similar to general population reference values. Correlation between the corresponding subscales of both instruments ranged between 0.59 (Social Role) and 0.78 (Physical Functioning). Both an exploratory and a theoretically driven confirmatory factor analysis provided further support for conceptual agreement of the scales.

**Conclusion:**

EORTC QLQ-C30 and PROMIS-29 showed similar scores and satisfactory agreement in conceptional and statistical analysis. This suggests that the underlying conceptualization of health is reasonably close. Hence, the development of score transformation algorithms or calibration of both instruments on common scales could prospectively increase the comparability of clinical and research PRO data collected with either instrument.

**Supplementary Information:**

The online version contains supplementary material available at 10.1007/s11136-022-03324-7.

## Purpose

Collecting patient-reported outcomes (PROs) to measure health-related quality of life (HRQOL) in clinical routine has become more common and accepted worldwide, as recent studies have shown that a systematic collection of PROs as a monitoring tool can improve overall survival and reduce hospitalization and emergency visits [1]. Furthermore, routine collection of PRO data improves patient‒physician communication and can lead to better outcomes [2–4].

PROs collected in clinical routine allow clinicians to compare outcomes of similar patient groups between countries and to improve overall health care quality [5]. Currently, over 3,900 PRO instruments have been developed and are available to measure HRQOL [6]; the greatest challenge for the upcoming years is to standardize PRO assessments with common underlying latent constructs such as depression, physical function, fatigue, etc.

Two PRO instruments that are widely used to assess HRQOL in a comprehensive manner are the Quality of Life Questionnaire-Core30 (QLQ-C30), developed by the European Organization for Research and Treatment Cancer (EORTC), and the PROMIS-29 as part of the Patient-Reported Outcome Measurement Information System (PROMIS). The QLQ-C30 was developed as a core instrument for patients with any cancer. Therefore, the instrument contains relevant items covering generic general health as well as items covering common symptoms following cancer therapy (e.g. nausea, appetite loss) [7]. The PROMIS-29 was developed to capture PROs for a wide range of chronic illnesses and disorders [8], and therefore covers only domains of general health (e.g. physical function, pain). Both instruments share the same definition of health as a multidimensional construct of dimensions such as physical function, mental health, social participation, sleep, and pain [7, 9]. Both are also based on the methodical and conceptual framework by Ware et al. [10, 11] to assess the health status of patients. A main difference between those two measures is the population they were developed for. Nevertheless, the PROMIS-29 has been successfully used in cancer populations [12–14] and is compared to the QLQ-C30 equivalent in terms of value and usefulness for patients and clinicians [15].

Both instruments are centrepieces of a wider framework of health assessment and have each been translated and validated in more than 40 languages. While PROMIS measures were developed using item response theory (IRT) methods, EORTC initially used methods of classical test theory for questionnaire development and recently adopted IRT to develop item banks for all QLQ-C30 domains. IRT allows a more flexible and efficient measurement of the targeted domains, e.g. using computer adaptive tests (CAT) or creating specific shortforms [16]. IRT is also used to score each domain measured on a standardized T-score metric, where 50 describes the mean (M) and 10 the standard deviation (SD). For both instruments, 50 represents the mean score in the general population in the US and Europe showing no significant differences between the population. [17] [18] [19] Using such a *T*-score metric makes similar domains from different instruments directly comparable [20].

One example that highlights the similarities and shared conception underlying both instruments is that the U.S. Food & Drug Administration (FDA) recommends both the EORTC and the PROMIS Physical Function scales in its Guidance Document “Core Patient-Reported Outcomes in Cancer Clinical Trials” [21]. As a first step to allow direct comparability of the results measured with the QLQ-C30 and PROMIS-29, the aim of this paper is to (1) descriptively assess the similarity of the PROMIS-29 and the EORTC QLQ-C30 at the domain and item levels, (2) empirically assess the agreement of comparable domain scores in terms of mean scores and correlations, and (3) assess whether a common underlying factor structure for both measures can be established. We expect that the mean scores of similar domains are reasonably close and that the correlation coefficients show a strong association (> 0.6) between convergent domains. The following exploratory factor analysis (EFA) and confirmatory factor analysis (CFA) will further investigate the underlying psychometric structure of both instruments.

## Methods

### Sample

PROMIS-29 and QLQ-C30 data were collected from patients visiting the breast cancer outpatient clinic at Charite – Universitaetsmedizin Berlin during routine diagnosis between November 2016 and March 2021. We included the first PRO assessment that was taken at the initial visit from 1,478 female ambulant patients for our analysis regardless of the breast desease (breast cancer, fibroadenom and other). To avoid missing responses, the digital survey did not allow skipping questions.

### Measures

The QLQ-C30 is a cancer-specific and core instrument in the library of EORTC instruments focussing on the general HRQOL and was first mentioned in 1986 [7, 22]. It comprises five functional scales, social (SF), emotional (EF), cognitive (CF), role (RF) and physical (PF), plus 8 symptom scales, pain (PA), fatigue (FA), dyspnoea (DY), sleep disturbance (SD), appetite loss (AL), constipation (CO), diarrhoea (DI) and nausea & vomiting (NV). Additionally, the financial status (FD), a global quality of life and global health scale (QOL and GLQ) as well as several symptom measures are collected. In total, the QLQ-C30 uses 30 items. Responses to each item are made on a four-point Likert scale (1 = “Not at all”, 2 = “A little”, 3 = “Quite a bit”, 4 = “Very much”) for Items 1–28 and a seven-point Likert scale (1 = “Very poor” to 7 = “Excellent”) for Items 28 and 29 (global health and quality of life) [7]. The recall period is not specified for the first 5 items, and all the following 25 items use a week as the recall period.

The PROMIS was funded in 2004 by the United States under the National Institute of Health (NIH) Roadmap for Medical Research Initiative [9] and since then, developed over 100 item banks covering different aspects of physical, mental and social health. The PROMIS-29 Profile—a 29-item instrument—combines short assessments of eight core constructs of HRQOL: physical function (PF), sleep disturbance (SD), pain interference (PI) and pain intensity (PIN), fatigue (FA), anxiety (AN), depression (DE) and ability to participate in social roles and activities (SRAA). The instrument includes 8 subscales and a five-point Likert scale where 3 scales (AN, DE, SRAA) use 5 = “Never” to 1 = ”Always” and 3 scales (PI, SD, FA) use 5 = “Not at all” to 1 = “Very much” [19]. The PF Likert scale ranges from 5 = “Without any difficulty” to 1 = “unable to do”. Pain intensity is measured on a 10-Point visual analogue scale (VAS) from 0 = “No pain” to 10 = “Worst pain imaginable”. All domains use a 7-day recall period, except for PF and SRAA. [8, 19]. Table 1 shows the scale and item content for both instruments.

**Table 1:** Domains and items of the PROMIS-29 and the EORTC QLQ-C30

PROMIS-29		QLQ-C30	
Domain	Item	Domain	Item
Physical function	Ability to do chores such as vacuuming or yard work	Physical function	Strenuous activities, like carrying a heavy shopping bag or a suitcase
	Ability to run errands and shop		Take a long walk
	Ability to go for a walk of at least 15 min		A short walk outside of the house
	Ability to go up and down stairs at a normal pace		Stay in bed or a chair during the day
			Help with eating, dressing, washing yourself or using the toilet
Anxiety	Feel fearful	Emotional function	Worry
	Hard to focus on anything other than anxiety		Feel tense
	Overwhelmed by worries		Feel depressed
	Feel uneasy		Feel irritable
Depression	Feel worthless		
	Feel helpless		
	Feel depressed		
	Feel hopeless		
Pain	Pain interferes with day-to-day activities	Pain	Had pain
	Pain interferes with work around the home		Pain interference with daily activities
	Pain interferes with ability to participate in social activities		
	Pain interferes with household chores		
	Pain scale		
Fatigue	Feel fatigued	Fatigue	Need to rest
	Trouble starting things because I am tired		Feel weak
	Average feeling of run-down		Tired
	Average fatigue		
Sleep	Sleep quality	Sleep	Trouble sleeping
	Sleep was refreshing		
	Problem with sleep		
	Difficulty falling asleep		
Social activity	Regular leisure activities with others	Role function	Doing either work or other daily activities
	Family activities that I want to do		Pursuing hobbies or other leisure time activities
	Usual work (including work at home)	Social function	Physical condition or medical treatment interfered with family life
	Activities with friends that I want to do		Physical condition or medical treatment interfered with social activities

### Analysis

We summarized the demographic characteristics of the sample*.* We then transformed the individual item responses for each domain to *T*-scores for both the QLQ-C30 and the PROMIS-29 using algorithms provided by the EORTC QLQ group and the German PROMIS National Center. Means and standard deviations for the PROMIS-29 and QLQ-C30 domain scores were computed. We compared these scores with general population reference data reported in the previous papers [17, 18]. We assessed correlations between all domains using the Pearson correlation coefficient. We defined a correlation > 0.6 as high and > 0.4 as moderate. [23] Furthermore, we performed an exploratory factor analysis (EFA) and confirmatory factor analysis (CFA) to evaluate the dimensional structure of both instruments.

The combined dataset was randomly split in half, resulting in an equal amount of *N* = 739 for EFA and CFA. We investigated the number of factors to extract in the EFA dataset by assessing eigenvalues using the Kaiser criterion. Since this tends to overestimate the number of factors to extract, we also performed a scree test and a parallel analysis. We performed ordinary least squares factor analysis based on the polychoric correlation matrix to account for the ordinal nature of the items and used oblimin rotation to account for the correlated nature of the constructs. We then compared multiple factor solutions exploratively by assessing the resulting patterns of factor loadings.

For the CFA, we estimated an a priori specified model based on our content analysis and the results from the EFA. For this model, we assigned items of the same construct from both the PROMIS-29 and QLQ-C30 to the correlated latent factors. To account for the ordinal nature of the responses, we used the WLSMV estimator. We report standardized model parameter estimates and assessed the comparative fit index (CFI), the root mean square error of approximation (RMSEA) and the standardized root mean square residual (SRMR) to evaluate model fit (criteria RMSEA < 0.08, CFI > 0.95, SRMR < 0.08 [24]). Modification indices were descriptively assessed to investigate potential misspecifications of the model.

Symptom measures of the QLQ-C30 as well as the FD, CF, NV and global scales were excluded a priori from the correlation analysis, EFA and CFA. FD was excluded as it is neither a measure of function nor a symptom. The symptoms and global scales were excluded since they are not covered by the PROMIS-29. We excluded the PROMIS Pain intensity item, as it is a numerical analogue scale not used in latent variable modelling.

All analyses were conducted with R version 4.0.2 software using the following packages: lavaan (0.6–8), nFactors (2.4.1), tidyr (1.1.2) and dplyr (1.0.3).

## Results

### Content comparison

The content assessment of both instruments showed a similar conceptualization of health on the domain as well as on the item level (Table 1). Each of the seven domains of the PROMIS-29 is assignable to one domain of the QLQ-C30. The PROMIS-29 domains AN and DE are linked to the EF domain of QLQ-30, and the SRAA domain combines the two QLQ-C30 domains RF and SF. A main difference between both instruments is the number of items used to measure a domain. Whereas the PROMIS-29 uses 4 items for each domain, the QLQ-C30 uses a different number of items to measure a single construct: for example, one item for SF, 3 items for FA and 5 items for PF (see Table 1).

In addition to the comparability of the content, the wording used in individual items further shows similarities between the two instruments. The items measuring depression both use the wording “feel depressed”/“felt depressed”, and for anxiety, both use “worry”/“worries”. Similar wording can also be seen for items in the domains FA, PI and SD. Above all, there are differences that are particularly evident within the spectrum of the domains. Looking at the domain PF, the QLQ-C30 items cover a wider range from very basic (eating, dressing), easy (short walk) and difficult tasks (long walk), whereas the spectrum of the PROMIS-29 does not cover basic tasks. The PROMIS-29 domain SL, however, covers a broader spectrum of sleep quality than the single item used in the QLQ-C30.

Looking at the overall wording items of the generic instrument, PROMIS-29 does not reflect any specific disease. Despite QLQ-C30 being developed as a cancer-specific instrument, no reference to a specific disease is made in item wording.

### Descriptive statistics

The mean age of the 1,478 female patients in the sample was 47.4 years (SD = 14.49, range 15–90), with 41.54% of the women being diagnosed with malignant and 26.86% with benign breast cancer. Most patients (65.97%) had a high school diploma or further education. Demographics are summarized in Table 2.

**Table 2:** Demographic characteristics of the sample

Patient characteristics (*N* = 1,478)		
Characteristics	No	%
*Age at enrolment*		
Mean		47.4
Range		15–90
*Age group*		
< 30	192	12.99%
30–40	312	21.11%
41–50	391	26.45%
51–60	309	20.91%
61–70	177	11.98%
> 70	97	6.56%
*Education group*		
High	975	65.97%
Middle	369	24.97%
Low	128	8.66%
not specified	6	0.41%
*Diagnose group*		
Mamma-CA	572	38.70%
Fibroadenoma	225	15.22%
Benign	397	26.86%
DCIS	42	2.84%
Second opinion	242	16.37%

Table 3 shows the *T*-scores of both instruments by domain. We observed no relevant deviation regarding HRQOL of our cohort from the general population, as the mean scores of all domains were close to 50 (M = 50, SD ± 10) [17, 18]). An exception is mental health, where we observed elevated levels of anxiety (PROMIS) and reduced levels of emotional functioning (EORTC). Overall, the mean *T*-scores of the corresponding domains of both measures show only small differences (< 5) between each other. More descriptives results (mean, standard deviation, normdata, kurtosis, skewness) of the included health domains can be found in the electronic supplement Table I.

**Table 3:** Results of PROMIS-29 and QLQ-C30 compared to norm data

		PROMIS-29		QLQ-C30	
		*T*-Score Mean (SD)	Norm	*T*-Score Mean (SD)	Norm
Mental health	Anxiety	56.19 (9.82)^d^	53.1		
	Depression	51.52 (8.94)^d^	52.7		
	Emotional function			44.17 (9.72)^a^	50.63
	Fatigue	47.85 (10.11)^d^	48.3	51.52 (10.15)^b^	51.17
Physical health	Physical function	51.17 (7.61)^c^	51.4	50.68 (9.74)^a^	48.33
	Pain Interference	48.79 (8.93)^d^	52.4	50.18 (9.03)^b^	50.84
	Sleep disturbance	50.43 (8.95)^d^	49.2	54.22 (8.41)^b^	50.96
Social health	Social roles and activities	54.04 (9.63)^c^	50.8		
	Role function			48.96 (9.04)^a^	49.13
	Social function			47.86 (8.76)^a^	49.93

^a^QLQ-C30 domains in T-score, 50 = population average, −/ + 10 SD worse/better than population average, i.e. higher values indicate better function

^b^QLQ-C30 domains in T-score, 50 = population average, −/ + 10 SD better/worse than population average, i.e. lower values indicate better function

^c^PROMIS domains in T-score, 50 = population average, −/ + 10 SD worse/better than population average, higher values indicate better function

^d^PROMIS domains in theta T-score, 50 = population average, −/ + 10 SD better/worse than population average, lower values indicate better function

### Correlation analysis

All PROMIS-29 domains correlate highly (> 0.6) with at least one other domain of the QLQ-C30. Most of the similar descriptive domains show the highest correlation to each other except for the QLQ-C30 domain RF, with a correlation to the corresponding domain of 0.59 and a higher correlation to PF and PI of the PROMIS-29 (0.68 and −0.63). As expected from the content analysis, the QLQ-C30 domain EF shows high correlations with the PROMIS-29 domains AN (−0.69) and DP (−0.70) and with FA (−0.62). All coefficients are summarized in Table 4.

**Table 4:**
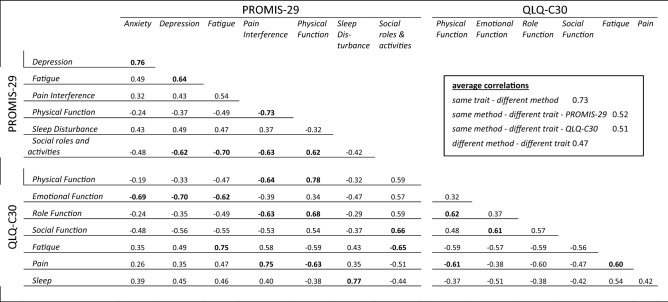
Correlation coefficients of the PROMIS-29 and QLQ-C30 domains, highlighted correlations > 0.6/ < −0.6 and multitrait-multimethod correlation matrix.

The multitrait-multimethod analysis showed a high average correlation of 0.73 for conceptually similar domains. The average correlation attributed to the method is similar, looking only at PRMIS-29 and QLQ-30, with 0.52 and 0.51, respectively. The average correlation for conceptually distinct domains that do not share the same method is lowest at 0.47.

### Exploratory factor analysis (EFA)

The EFA resulted in 6 eigenvalues greater than 1 (19.95, 5.08, 3.02, 1.85, 1.39, 1.14), indicating 6 factors to extract according to the Kaiser criterion. Parallel analysis also suggested 6 factors. An inspection of the scree plot suggests 3–5 factors. We, therefore, estimated four different EFA models with 3 to 6 factors.

We found the EFA with 5 factors to be interpretable as the most meaningful. These 5 factors were categorized as Physical Function, Mental Health, Fatigue, Pain and Sleep. These factors explain 72% of the total variance. All items of both instruments related to pain and sleep load on the same factor. All PROMIS-29 domains and associated items show the highest loading to one modelled factor with loadings of 0.6/−0.6 and higher/lower, respectively, except all items of the SRAA domain, which have loadings between −0.56 and −0.51 to the factor Physical Function. Eight out of 19 items of the QLQ-C30 did show a relevant loading for a single factor, and six showed a moderate or single relevant loading assignable to one factor. Items of the QLQ-C30 domain EF and SF have cross-loadings to several factors, whereas two QLQ-C30 EF items show the highest loadings on the factor Fatigue (0.51, 0.49) and two on the factor Mental Health (0.55, 0.51). All factor loadings of the Exploratory Factor Analysis with 5 factors can be found in the electronic supplement Table II.

### Confirmatory factor analysis (CFA)

**Fig. 1:**
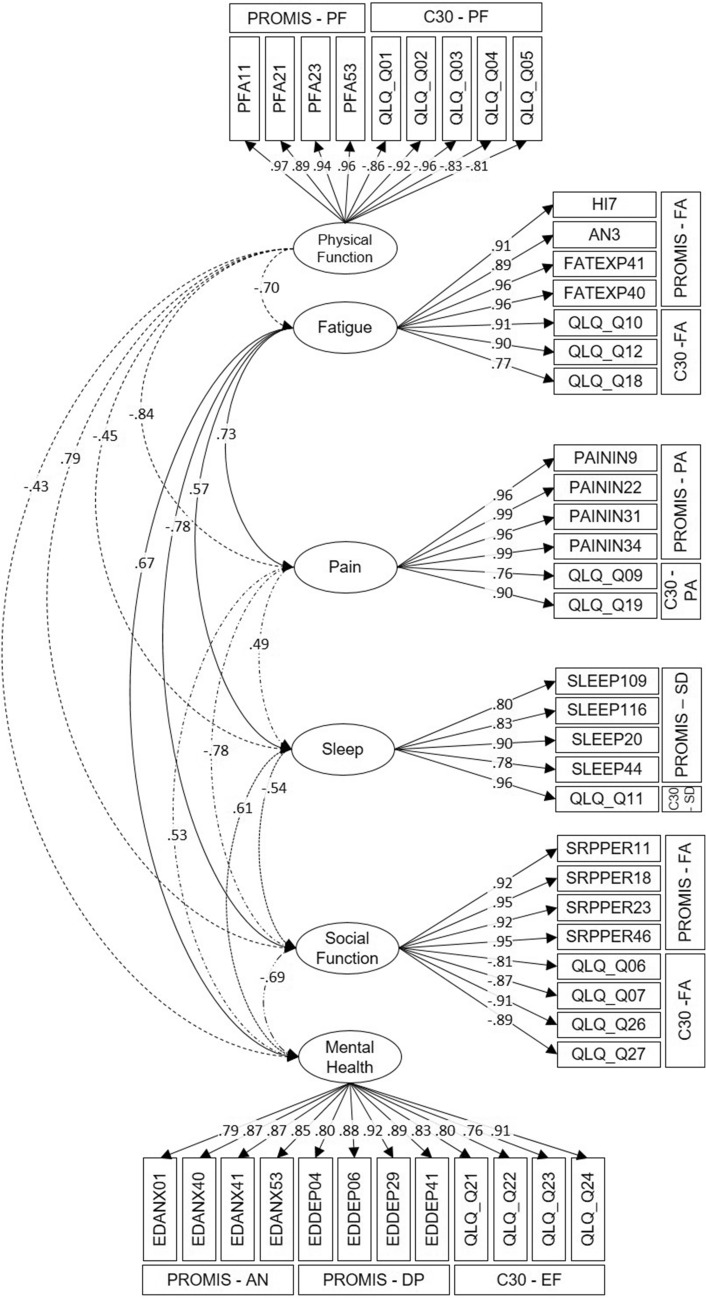
Confirmatory factor analysis—loadings of all items and factors to one another

Content analysis showed 7 domains with corresponding items from both measures. Based on the EFA, we modelled anxiety and depression as common factors. Figure 1 shows the resulting CFA model, which had a satisfactory fit of the data according to RMSEA = 0.074, CFI = 0.968 and SRMR = 0.074. Overall, we found that all items had high loadings (> 0.6) on the hypothesized factors and that correlation between factors was as expected. The top modification indices suggest additional common variance not explained by our hypothesized model between the QLQ-C30 question 10 (“Did you need to rest?”) and the physical function and pain factors. Additionally, the PROMIS-29 item “I found it hard to focus on anything other than my anxiety” showed additional relations to factors of fatigue, social role, physical function and pain. Since the model fit was satisfactory, we did not model these additional associations.

## Discussion

This paper focuses on the comparability of the two instruments QLQ-C30 and PROMIS-29 and their similar domains to prospectively increase the comparability of clinical and research PRO data collected with either instrument. In this study, we descriptively assessed the underlying HRQOL model of the PROMIS-29 and QLQ-C30 before we evaluated the similarity of both instruments empirically in a sample of 1,549 patients visiting the outpatient clinic for breast cancer and assessed whether a common underlying factor structure for both measures could be established. Our results demonstrate that the underlying conceptualization of health is comparable in both instruments.

First, we found a descriptive similarity of both instruments on the domain as well as on the item level indicated by similar wording in domain titles and item descriptions. Despite the different patient population backgrounds of the two instruments—cancer patients in clinical trials vs. patients with chronic diseases—there are structural similarities in the choice of health domains, implying a similar understanding of health. This finding was expected because both are core instruments within their frameworks to measure general health.

Second, we demonstrated this similarity empirically by comparing the *T*-scores and evaluating the correlation coefficients of the subscales from both measures. It could be shown that subscales with similar descriptive content show similar *T*-scores and a stronger correlation than others. It can also be assessed that the domains PF and PI of the PROMIS-29 both show a strong correlation to the QLQ-C30 domains PF, RF and PA, indicating a strong association between those dimensions. The highest correlation was observed between the corresponding scales, but this finding shows that factors such as pain are interrelated or dependent with the outcome of factors such as PF and RF, which is particularly relevant for the interpretation of the results in practice. These findings are in line with the previous research [25, 26]. We also compared the *T*-scores to data from the general population and showed that they are similar, indicating that the results of our paper are not disease specific. This similarity between the outcomes of our sample and the general population is expected since data were collected prior to treatment and final diagnosis. Therefore, we would not expect major differences in the general population. What can be expected is a slightly higher score in anxiety compared to norm data due to upcoming diagnosis and/or therapy decisions. This was measured well by the PROMIS-29. Differences between the two scores may be due to the different underlying norm samples (EU and US) and the measurement accuracy of the instruments and subscales. For example, SD is measured with a single item by the QLQ-C30 and four items by the PROMIS-29.

Based on the conceptual overlap between the two instruments, we empirically developed a 5 factor model in an EFA framework. Unlike previous papers, our focus was not on the validation of the instruments and their subscales in a certain population. We focussed on analysing whether these two measures are part of a greater model, hence using the same latent constructs to measure HRQOL. For the PROMIS-29 items, this model shows that 24 of 28 items can be assigned to one of the factors. For the QLQ-C30, only 8 out of 19 can be clearly assigned to one factor. An assignment of the other items or even the domain to one single construct was not always clear, which is also the case in a model with fewer factors. Similar results were found in earlier research [26]. Taking into account the appropriate fit of a theoretically derived model in the CFA, it can be assumed that the PROMIS-29 and QLQ-C30 share a similar conceptional model of health as perceived by patients. However, in both the EFA and CFA, some theoretically distinct constructs were inseparable or showed high correlations. For example, Social Function and Physical Function constituted a single factor in the EFA and were highly correlated (*r* = 0.79) in the CFA. This suggests less than perfect discriminant validity.

Sharing the same metric is a huge advantage in comparing the outcome measured with two different instruments, the exchange of results, the clinical applicability and the reduction of the ambiguity of the interpretation [20]. Although both instruments share the same metric, the value 50 does not correspond to the 50 of the other instrument, since they have been calculated based on different patient populations (European for QLQ-C30 and US for PROMIS-29). However, as our and other research [18] showed, differences to the mean are minor, and comparison of scores is possible, with the general advantage outweighing the minor differences [20]. However, differences in the calibration samples could potentially mask real differences. Therefore, to allow precise comparisons between assessments made with both instruments, a common measurement model based on the same population is necessary.

### Strengths and limitations

A major limitation of this study is that the sample is a convenience sample. This study evaluated data only from one sex and only from patients with breast-related diseases. Therefore, we cannot generalize our findings to men or patients with other diseases. However, since all domains of both scores in our sample show no or only little deviation compared with the general population, it can be assumed that this results in a general validity.

A further limitation is derived from the number of items used to measure each construct. Since both measures are streamlined for rapid, individual, and comprehensive HRQOL, assessment results scoring leads to limited precision. To develop common measurement models across both instruments, it would be necessary to include enough items to adequately represent the full range of each construct with a reasonable measurement precision.

Finally, we did not develop conversion tables between PROMIS-29 and EORTC QLQ-C30 since both questionnaires only include very few items of each item bank, which are likely not reflecting the broad range of the underlying constructs and therefore score conversion could be biassed.

Despite these limitations, our work has strengths, such as a large dataset with over 1,500 patients in a relevant population. Furthermore, the results from this sample have been compared to the general population, showing no major differences between both groups prior to treatment.

## Conclusion

The EORTC QLQ-C30 and PROMIS-29 are two instruments that measure HRQL with a focus on different populations, which is why the QLQ-C30 covers a broader spectrum of dimensions and the instruments shall not be seen as redundant. Our research showed similar scores and satisfactory agreement in conceptual and statistical analysis between the two instruments. This suggests that the underlying conceptualization of health is reasonably close. Hence, the development of score transformation algorithms or calibration of overall HRQOL domains of both instruments on common scales could prospectively increase the comparability of clinical and research PRO data collected with either one and increase flexibility when choosing an instrument. The datasets analysed during the current study are not publicly available due to data privacy regulations but can be made available from the corresponding author on reasonable request.

## Supplementary Information

Below is the link to the electronic supplementary material.

Supplementary file1 (PDF 87 KB)

## Data Availability

The datasets analysed during the current study are not publicly available due to data privacy regulations but can be made available from the corresponding author on reasonable request.
