# Supplementary material for: PROMIS-29 and EORTC QLQ-C30: an empirical investigation towards a common conception of health
Source: Qual Life Res. 2023 Jan 9;32(3):749–58. doi: 10.1007/s11136-022-03324-7 (PMC9992045; doi:10.1007/s11136-022-03324-7)
Supplement: Supplementary file 1 — Supplementary file1 (PDF 87 KB) [file 11136_2022_3324_MOESM1_ESM.pdf]

Electronic supplement to publication:

PROMIS-29 and EORTC QLQ-C30 – an empirical investigation towards a common conception of health

Table I: Descriptives (mean, standard deviation, normdata, kurtosis, skewness) of the included health domains

		PROMIS-29				QLQ-C30				
		T-Score Mean (SD)	Norm	Kurtosis	Skewness	Raw-Score Mean (SD)	T-Score Mean (SD)	Norm	Kurtosis	Skewness
Mental Health	Anxiety	56.19 (9.82)	53.1	-0.49	0.06					
	Depression	51.52 (8.94)	52.7	-0.45	0.42					
	Emotional function					59.99 (27.53)	44.17 (9.72)	50.63	-0.77	-0.38
	Fatigue	47.85 (10.11)	48.3	-0.18	0.43	29.66 (26.94)	51.52 (10.15)	51.17	-0.25	0.78
Physical Health	Physical function	51.17 (7.61)	51.4	0.06	-1.03	87.02 (18.34)	50.68 (9.74)	48.33	2.78	-1.77
	Pain Interference	48.79 (8.93)	52.4	0.01	0.95	23.44 (27.48)	50.18 (9.03)	50.84	0.53	1.17
	Sleep disturbance	50.43 (8.95)	49.2	-0.03	0.08	34.89 (33.91)	54.22 (8.41)	50.96	-0.86	0.56
Social Health	Social roles and acti	54.04 (9.63)	50.8	-0.60	-0.57				0.64	-1.27
	Role function					85.30 (26.23)	48.96 (9.04)	49.13	2.77	-1.90
	Social function					78.71 (28.10)	47.86 (8.76)	49.93		

Table II: Results of the Exploratory Factor Analysis with 5 factors, factor loadings are standardized

			oblimin 5					
			PF	Mental	Fatigue	Pain	Sleep	
	Item Domain	Item-ID	Factor1	Factor2	Factor3	Factor 4	Factor5	communlity
1	PROMIS PF	PROMIS29_PFA11	-0,61	-0,06	0,01	-0,32	-0,06	0,80
2	PROMIS PF	PROMIS29_PFA21	-0,71	0,08	-0,02	-0,18	-0,12	0,78
3	PROMIS PF	PROMIS29_PFA23	-0,80	0,00	0,04	-0,15	-0,09	0,84
4	PROMIS PF	PFA53	-0,73	-0,04	-0,06	-0,21	-0,01	0,87
5	PROMIS Anxiety	PROMIS29_EDANX01	-0,17	0,81	0,06	0,09	0,05	0,73
6	PROMIS Anxiety	EDANX40	-0,02	0,91	-0,12	0,11	0,02	0,80
7	PROMIS Anxiety	PROMIS29_EDANX41	-0,01	0,87	-0,08	0,09	0,07	0,78
8	PROMIS Anxiety	EDANX53	-0,10	0,82	0,07	0,05	0,09	0,79
9	PROMIS Depression	PROMIS29_EDDEP04	0,26	0,60	0,05	-0,05	0,03	0,54
10	PROMIS Depression	PROMIS29_EDDEP06	0,21	0,72	0,09	-0,02	0,07	0,77
11	PROMIS Depression	PROMIS29_EDDEP29	0,12	0,69	0,20	-0,06	0,11	0,79
12	PROMIS Depression	PROMIS29_EDDEP41	0,27	0,73	0,03	-0,10	0,11	0,76
13	PROMIS Fatigue	PROMIS29_HI7	0,13	0,17	0,63	0,03	0,08	0,74
14	PROMIS Fatigue	PROMIS29_AN3	0,27	0,12	0,60	-0,06	0,06	0,69
15	PROMIS Fatigue	PROMIS29_FATEXP41	0,07	0,15	0,68	-0,01	0,12	0,73
16	PROMIS Fatigue	PROMIS29_FATEXP40	0,09	0,14	0,69	0,04	0,08	0,77
17	PROMIS Sleep	PROMIS29_SLEEP109	-0,01	-0,02	-0,06	-0,10	0,94	0,75
18	PROMIS Sleep	PROMIS29_SLEEP116	0,04	-0,05	0,08	-0,06	0,80	0,65
19	PROMIS Sleep	PROMIS29_SLEEP20	-0,06	0,09	-0,03	0,05	0,88	0,82
20	PROMIS Sleep	PROMIS29_SLEEP44	0,04	0,16	-0,10	0,03	0,72	0,60
21	PROMIS Social	PROMIS29_SRPPER11_CAPS	-0,51	-0,31	-0,24	-0,04	-0,01	0,72
22	PROMIS Social	PROMIS29_SRPPER18_CAPS	-0,51	-0,28	-0,25	-0,04	-0,01	0,71
23	PROMIS Social	PROMIS29_SRPPER23_CAPS	-0,53	-0,27	-0,32	-0,02	-0,02	0,80
24	PROMIS Social	PROMIS29_SRPPER46_CAPS	-0,56	-0,30	-0,29	0,01	0,02	0,79
25	PROMIS Pain	PROMIS29_PAININ9	0,02	0,14	-0,05	0,90	0,03	0,90
26	PROMIS Pain	PROMIS29_PAININ22	0,23	0,10	-0,06	0,76	0,01	0,87
27	PROMIS Pain	PROMIS29_PAININ31	0,28	0,11	0,00	0,64	0,06	0,83
28	PROMIS Pain	PROMIS29_PAININ34	0,23	0,06	-0,04	0,73	0,07	0,87
29	EORTC PF	EORTCQLQC30_Q01	0,61	-0,08	0,10	0,15	0,14	0,66
30	EORTC PF	EORTCQLQC30_Q02	0,68	-0,17	0,04	0,19	0,18	0,78
31	EORTC PF	EORTCQLQC30_Q03	0,75	-0,08	0,05	0,09	0,07	0,73
32	EORTC PF	EORTCQLQC30_Q04	0,47	-0,09	0,26	0,19	0,04	0,59
33	EORTC PF	EORTCQLQC30_Q05	0,68	0,19	0,00	0,04	-0,13	0,55
34	EORTC RF	EORTCQLQC30_Q06	0,45	0,02	0,20	0,33	0,05	0,74
35	EORTC RF	EORTCQLQC30_Q07	0,39	-0,03	0,24	0,39	-0,02	0,70
36	EORTC PA	EORTCQLQC30_Q09	-0,04	-0,09	0,12	0,71	0,07	0,57
37	EORTC PA	EORTCQLQC30_Q19	-0,05	-0,04	0,17	0,84	0,06	0,83
38	EORTC SL	EORTCQLQC30_Q11	0,00	-0,03	0,08	0,09	0,83	0,81
39	EORTC FA	EORTCQLQC30_Q10	0,26	-0,11	0,48	0,36	-0,01	0,76
40	EORTC FA	EORTCQLQC30_Q12	0,18	-0,10	0,59	0,24	0,20	0,84
41	EORTC FA	EORTCQLQC30_Q18	-0,04	-0,18	0,81	0,18	0,10	0,76
42	EORTC EF	EORTCQLQC30_Q21	-0,15	0,37	0,51	0,05	0,10	0,62
43	EORTC EF	EORTCQLQC30_Q22	-0,23	0,55	0,31	0,15	0,07	0,61
44	EORTC EF	EORTCQLQC30_Q23	-0,11	0,37	0,49	0,05	0,02	0,53
45	EORTC EF	EORTCQLQC30_Q24	-0,13	0,51	0,45	0,07	0,15	0,78
48	EORTC SF	EORTCQLQC30_Q26	0,18	0,34	0,30	0,19	0,02	0,60
49	EORTC SF	EORTCQLQC30_Q27	0,24	0,28	0,37	0,22	-0,05	0,68
		Correlation with Factor1	1	0,25	0,47	0,67	0,31	
		Correlation with Factor2		1	0,48	0,26	0,42	
		Correlation with Factor3			1	0,47	0,46	
		Correlation with Factor4				1	0,42	
		Correlation with Factor5					1	
